# An Investigation Into the Role of Osteocalcin in Human Arterial Smooth Muscle Cell Calcification

**DOI:** 10.3389/fendo.2020.00369

**Published:** 2020-06-10

**Authors:** Sophie A. Millar, Stephen G. John, Christopher W. McIntyre, Vera Ralevic, Susan I. Anderson, Saoirse E. O'Sullivan

**Affiliations:** ^1^Division of Medical Sciences & Graduate Entry Medicine, School of Medicine, Royal Derby Hospital, University of Nottingham, Derby, United Kingdom; ^2^Department of Renal Medicine, Royal Derby Hospital, Derby, United Kingdom; ^3^London Health Sciences Centre, London, ON, Canada; ^4^Division of Physiology, Pharmacology and Neuroscience, School of Life Sciences, Queen's Medical Centre, University of Nottingham, Nottingham, United Kingdom

**Keywords:** osteocalcin, calcification, CKD, vascular, mineralization

## Abstract

Osteocalcin (OCN) is a bone-derived protein that is detected within human calcified vascular tissue. Calcification is particularly prevalent in chronic kidney disease (CKD) patients but the role of OCN in calcification, whether active or passive, has not been elucidated. Part 1: The relationship between OCN, CKD and vascular calcification was assessed in CKD patients (*n* = 28) and age-matched controls (*n* = 19). Part 2: *in vitro*, we analyzed whether addition of uncarboxylated osteocalcin (ucOCN) influenced the rate or extent of vascular smooth muscle cell (VSMC) calcification. Human aortic VSMCs were cultured in control media or mineralisation inducing media (MM) containing increased phosphate with or without ucOCN (10 or 30 ng/mL) for up to 21 days. Markers of osteogenic differentiation and calcification were determined [alkaline phosphatase (ALP) activity, total intracellular OCN, Runx2 expression, α-SMA expression, alizarin red calcium staining, and calcium quantification]. Part 1 results: In our human population, calcification was present (mean age 76 years), but no differences were detected between CKD patients and controls. Plasma total OCN was increased in CKD patients compared to controls (14 vs. 9 ng/mL; *p* < 0.05) and correlated to estimated glomerular filtration rate (*p* < 0.05), however no relationship was detected between total OCN and calcification. Part 2 results: *in vitro*, ALP activity, α-SMA expression and calcium concentrations were significantly increased in MM treated VSMCs at day 21, but no effect of ucOCN was observed. Cells treated with control media+ucOCN for 21 days did not show increases in ALP activity nor calcification. In summary, although plasma total OCN was increased in CKD patients, this study did not find a relationship between OCN and calcification in CKD and non-CKD patients, and found no *in vitro* evidence of an active role of ucOCN in vascular calcification as assessed over 21 days. ucOCN appears not to be a mediator of vascular calcification, but further investigation is warranted.

## Introduction

Vascular calcification is a known major risk factor for mortality and morbidity, particularly within chronic kidney disease patients, and is an independent risk factor for cardiovascular disease (1–3). CKD-mineral bone disorder (CKD-MBD) entails derangements in mineral metabolism, bone remodeling abnormalities, and accelerated medial and intimal calcification, which worsens under haemodialysis (4). In stage 5 CKD patients, calcification in particular is driven by vascular apoptosis and osteogenic differentiation triggered by increased phosphate levels (4). Calcification involves the progressive deposition of calcium within vessels, reducing elasticity and impairing cardiovascular function by promoting mechanical failure (5). As the human population continues to age and increase longevity, the consequences of such diseases are further pronounced. Long believed to be a passive part of aging “wear and tear,” vascular calcification is now considered an active, cell-mediated complex process that is a regulated form of extracellular matrix bio-mineralisation but is not yet fully understood (5, 6).

In bone, bio-mineralisation occurs via endochondral ossification or membranous ossification programmed by chondrocytes and osteoblasts, initiated by matrix vesicles whose function is nucleation and growth of calcium crystals. Vascular smooth muscle cells (VSMCs) can trans-differentiate into osteoblast-like cells displaying osteogenic fingerprints generally characterized by a decrease in smooth muscle cell markers (e.g., α-SMA, SM-MHC) and an increase in osteogenic markers such as alkaline phosphatase (ALP), Runx2, SOX9, and osteocalcin (6, 7). In a remarkably similar way to bone, differentiated VSMCs demonstrate hydroxyapatite production and mineralisation (6). Hydroxyapatite crystals form within matrix vesicles secreted from the membranes of osteoblasts, odontoblasts, and chondrocytes (8). These buds provide a nidus for calcium, phosphate and mineral nucleation which is then deposited in the extracellular matrix between collagen fibrils (8). This active osteogenic process can be triggered by oxidative stress, oxylipids, phosphate, inflammatory oxylipids, and oxLDL (6).

Osteocalcin (OCN) is the most prominent non-collagenous protein found in the bone extracellular matrix, predominantly produced by osteoblasts and can be found in the circulation following bone resorption (9, 10). Osteocalcin has three main forms, carboxylated osteocalcin (cOCN), undercarboxylated osteocalcin (unOCN) and uncarboxylated osteocalcin (ucOCN). The carboxylation process is vitamin K dependent and induces a high affinity of osteocalcin for the calcium ions present in hydroxyapatite. OCN is additionally expressed by differentiated osteoblast-like VSMCs (7, 11). Interestingly, it has been shown that OCN is not required for bone mineralisation in mice (12, 13). It has been reported that OCN may delay nucleation and growth of hydroxyapatite in pig bone, and if this is also the case in differentiated VSMCs, OCN may be viewed as a vascular calcification inhibitor (14). Embedded OCN in calcified vascular regions has been positively correlated to the extent of vascular calcification in humans, but circulating concentrations have had conflicting reports (15). There has been very little experimental evidence documented on the role of OCN in calcification, and none to date in human cells nor using ucOCN. In mice, it has been demonstrated that OCN stimulates glucose utilization and promotes VSMC mineralisation and osteogenic differentiation, in particular through HIF-1α activation (16).

Investigation of OCN in human VSMCs is required to clarify its physiological importance. We measured circulating concentrations of total OCN in chronic kidney disease patients and in controls, and analyzed this alongside vascular calcification data. *In vitro*, we hypothesized that the addition of ucOCN at physiological and pathophysiological concentrations to human aortic VSMCs may increase the speed or extent of osteogenic calcification.

## Materials and Methods

### Patients

Investigations were performed on baseline data from a single-center cohort of blood pressure-controlled hypertensive CKD patients (*n* = 28) and age-matched controls (*n* = 19) (see Table 1 for patient demographics) (17, 18). The study was originally approved by the Local Regional Ethics Committee and all patients gave informed consent. Plasma levels of osteocalcin (OCN) were measured using a commercially available assay (Milliplex MAP Human Bone Magnetic Bead Panel Cat no HBNMAG-51K, MerckMillipore). Multislice computed tomography (MSCT) was used to quantify calcification. A standardized section of the superficial femoral artery (SFA), 20 cm above the tibial plateau, 5 cm in length was imaged in *n* = 20 2.5 mm slices per person; care was taken to ensure that none of the slices overlap. Each slice was scored individually and a calcification score was generated. Calcification was considered to be present if an area ≥1 mm displayed a density >130 Hounsfield units (19). Validation studies confirmed that the scoring technique is highly reproducible. Inter-observer reproducibility between the investigator and a consultant radiologist was assessed in a 1-in-20 sample. The intraclass correlation was 1 [confidence interval (CI) 1 to 1] and the CoV was 3.9%. Repeatedly scored scans showed an intra-observer intraclass correlation of 1 (CI 1 to 1) and a CoV of 2.4%. Carotid-femoral pulse wave velocity (PWVcf) was assessed by ECG-gated applanation tonometry using a SphygmoCor^®^ (AtCor Medical Pty Ltd., Australia). Non-invasive continuous pulse wave analysis was used to determine hemodynamic variables, described previously (17).

**Table 1:** Population characteristics.

	**Non-CKD controls (mean ± SD)**	**CKD patients (mean ± SD)**	***T*-test (*p*-value)**
	*N* = 19^*^	*N* = 29^*^	
Gender (F/M)	7/12	13/16	
Age (years)	76 ± 4.8	76 ± 4.4	NS
BMI (kg/m^2^)	25.90 ± 4.00	25.40 ± 3.50	NS
Mean blood pressure (mmHg)	103.50 ± 9.80	104.00 ± 12.30	NS
Serum creatinine (μmol/L)	73.80 ± 21.00	143.70 ± 56.60	<0.0001
eGFR (mL/min per 1.73m^2^)	93.11 ± 35.85	42.97 ± 13.73	<0.0001
Urine protein/creatinine ratio	0.11 ± 0.04	0.31 ± 0.42	<0.05
Hemoglobin (g/dL)	13.98 ± 1.69	12.76 ± 1.74	<0.01
Urea (mmol/L)	5.81 ± 1.79	9.67 ± 2.95	<0.0001
Corrected calcium (mmol/L)	2.37 ± 0.09	2.33 ± 0.08	NS
Phosphate (mmol/L)	1.07 ± 0.16	1.09 ± 0.14	NS
PWVcf (m/s)	13.63 ± 2.73	12.93 ± 2.40	NS
Calcium score^*a*^	2.50 (0.00–29.00)	3.50 (0.00–30.75)	NS
Calcification density^*a*^	2 (0–3)	2 (0–4)	NS

*BMI, body mass index; eGFR, estimated glomerular filtration rate; PWVcf, carotid-femoral pulse wave velocity; NS, not significant*.

a *Median and interquartile range*;

* *n = 16 for non-CKD, n = 22 for CKD patients with valid calcium score data*.

### Cell Culture

Primary human aortic smooth muscle cells (HASMCs) were obtained from PromoCell (UK) and maintained at 37°C in a humidified incubator supplemented with 5% CO_2_ in commercially available smooth muscle cell growth media (PromoCell, UK). Cells were used at passage 4 and 5. Human osteoblasts (HOBs) were originally isolated from human femoral head trabecular bone and have been characterized previously (20–22). HOBs were cultured in osteoblast growth media (PromoCell, UK) and maintained as above. All experiments were performed in confluent cells. After experimental treatments, cell media and cell lysates were collected and frozen at −80°C prior to analysis.

### Osteocalcin

**Figure 1:**
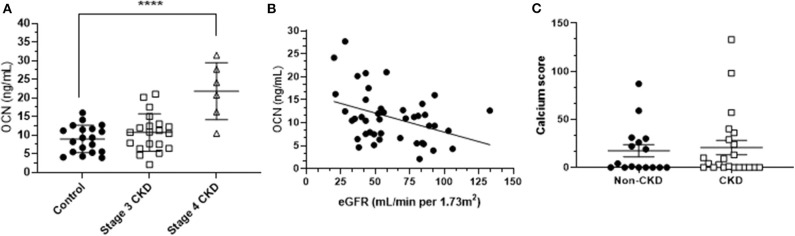
**(A)** Mean (± SD) osteocalcin (OCN) concentrations of stage 3 CKD patients, stage 4 CKD patients and age-matched controls. Differences were assessed by one-way ANOVA. ^****^ indicates *p* < 0.001. **(B)** The osteocalcin-eGFR (estimated glomerular filtration rate) relationship, assessed by Spearman's correlation (*r* = −0.32; *p* < 0.05). **(C)** Mean (± SD) calcification scores of CKD patients and age-matched controls.

Human fully uncarboxylated osteocalcin [ucOCN; amino acids 1–49, (Glu17,21,24)] was purchased from AnaSpec Inc. CA (AS-65307). The same batch of ucOCN has been previously shown to be biologically active in vascular cells in our previous work (23). Additionally, samples of ucOCN were routinely measured by duoset ELISA (see osteocalcin quantification section below) to monitor stability and consistent concentration throughout the experimental period. We used ucOCN as it has previously been deemed the “active” form of osteocalcin within the circulation and is present in higher concentrations than cOCN. Based on our patient data (Figure 1B), 10 and 30 ng/mL concentrations of ucOCN were chosen.

### Calcification Experiments

For inducing calcification, cells were grown in commercially available mineralisation media (PromoCell, UK; C-27020) for up to 21 days. Due to its proprietary nature the exact media composition is not disclosed, however it was communicated by personal email with PromoCell to contain elevated phosphate concentrations similar to those used in the published literature to induce calcification. Cells were treated with or without ucOCN (10 or 30 ng/mL). Media and ucOCN were replaced every 3rd day. All experiments were performed independently at least three times, with a minimum *n* = 2 for each condition at each time point, with the exception of the HOBS experiments which were performed twice.

### Osteocalcin, MMP-3 and IL-1β, and Quantification

Total human intracellular and extracellular osteocalcin was measured using an enzyme linked immunosorbent (ELISA) duoset assay (R&D systems, DY1419). Whole cell lysates and spent cell culture media were collected on days 0, 6, 12 18, and 21. Secreted human total matrix metalloproteinase-3 (MMP-3) and interleukin-1β (IL-1β) were measured using ELISA kits (R&D systems, DY513 and DY201). Assays were performed according to manufacturer's instructions.

### Total Protein Quantification

A bicinchoninic acid protein (BCA) assay was performed to quantify the total protein content in cell lysates at days 0, 6, 12, 18, and 21 (24). The BCA working reagent was prepared by mixing BCA solution with copper (II) sulfate pentahydrate 4% solution (Sigma-Aldrich, UK) at a 50:1 ratio. Protein concentrations of samples were determined by interpolation against a bovine serum albumin standard curve.

### Alizarin Red Staining and Calcium Quantification

Alizarin Red, or 1,2-dihydroxyanthraquinone was used to stain hydroxyapatite mineralized matrixes in cell monolayers producing a red-orange color. Alizarin Red powder (Sigma Aldrich) was dissolved in dH_2_O to make a 40mM solution, and pH adjusted to 4.1–4.3 with 0.5% ammonium hydroxide. Cells were fixed with 10% (v/v) formaldehyde (Sigma Aldrich) at room temperature for 15 min. The monolayers were then washed twice with excess dH_2_O. Alizarin Red solution was then added to each well and incubated at room temperature for 20 min. The unincorporated dye was then removed and the plates were washed 4 times with excess dH_2_O. To extract and quantify the incorporated dye, 10% (v/v) acetic acid was added to each well. The cell layer mixture in acetic acid was then collected into eppendorfs, vortexed, and overlaid with mineral oil. The eppendorfs were heated to 85°C for 10 min and transferred to ice to cool. The samples were centrifuged at 20,000 × g for 15 min and the supernatants removed and neutralized with ammonium hydroxide (10% v/v). Colorimetric detection was then carried out at 405 nm and data expressed as absorbance.

Calcium content was measured using a calcium detection assay kit (Abcam, ab102505) according to manufacturer's instructions. Briefly, cells were decalcified overnight with 0.6 M hydrochloric acid (HCL). The calcium contents of the supernatants were then quantified using the 0-cresolphthalein method in which a chromogenic complex is formed between calcium ions and 0-cresolphthalein and then measured at 575 nm using a spectrophotometric plate reader (25). Calcium quantification was performed on days 0 and 21.

### Alkaline Phosphatase (ALP) Activity

ALP activity was measured using an ALP detection assay kit (Abcam, ab83369) according to manufacturer's instruction. Briefly, p-nitrophenyl phosphate (pNPP) was used as a phosphatase substrate which turns yellow when dephosphorylated by ALP and absorbance was measured at 405 nm using a spectrophotometric plate reader. ALP activity was measured on days 0, 6, 12, 18, and 21.

### α-SMA, Runx2, and Sox9 Protein Expression

Cell lysate supernatants were collected and protein samples (10 μg/lane) were resolved by electrophoresis on 10% Mini-protean TGX precast gels (Bio-Rad Laboratories, Inc., UK). The proteins were wet transferred to a nitrocellulose membrane. Protein bands were visualized by staining with Ponceau S stain and imaged to quantify total lane protein as previously described (26). Membranes were then incubated in blocking buffer followed by incubation with either rabbit anti-human smooth muscle alpha actin (Abcam, ab32575, 1:2,500 dilution), goat anti-human Runx2 (R&D systems, AF2006, 1:2,000 dilution), or goat anti-human Sox9 (R&D systems, AF3075, 1:400 dilution) overnight at 4°C. The membrane was then washed and incubated for 1.5 h at room temperature with alkaline phosphatase conjugated anti-rabbit secondary antibody (Sigma, Catalog No. A3937, 1:25000 dilution in 3% marvel in TBST) or anti-goat secondary antibody (Abcam, ab97097, 1:5,000 dilution in 3% marvel in TBST). Immunoreactive bands were visualized by chemiluminescence (Bio-Rad Immun-Star™ AP Substrate Pack #1705012). Protein bands were visualized using the ChemiDoc™ MP Imaging system with Image Lab™ software (Bio-Rad). Proteins were normalized to total lane protein as determined by Ponceau S staining.

### Statistical Analysis

For the population data, univariate comparisons of continuous variables between CKD patients and non-CKD controls were performed using parametric or non-parametric (Mann-Whitney) *t*-tests with or without Welch's correction depending on distribution and variance as appropriate. A one-way ANOVA was used to assess differences in OCN concentrations between controls, CKD stage 3, and CKD stage 4 patients correcting for multiple comparisons with Dunnett's multiple comparison test. Spearman's correlation tests were performed to assess the relationships between OCN and other biological measurements including estimated glomerular filtration rate (eGFR) and cardiovascular parameters. Data are presented as means and standard deviation (SD) for parametric data, and median and interquartile range for non-parametric data are presented. For the *in vitro* data, two-way ANOVAs were used to assess differences between groups using day and treatment as factors for ALP activity, OCN quantification, MMP-3 quantification, IL-1β quantification and total protein quantification. One-way ANOVAs were used to assess differences between groups for Runx2, and α-SMA quantification, calcium quantification, and alizarin red staining quantification. Data are presented as means and standard error of the mean (SEM). Multiple comparisons were adjusted for by Dunnett's statistical hypothesis test. All statistical analyses were performed using Prism 8 for Windows (Version 8.01, GraphPad Software Inc.). *P*-values were considered significant at *p* < 0.05.

## Results

### OCN Concentration Is Increased in Stage 4 CKD Patients

The clinical characteristics of CKD patients and age-matched non-CKD controls are summarized in Table 1. CKD patients (stages 3 and 4, *n* = 29) had significantly higher serum creatinine and urinary protein to creatinine ratio and significantly lower eGFR and hemoglobin compared to controls (*n* = 19), as expected (Table 1). Other demographics and clinical parameters were similar between the two groups.

Mean plasma total OCN concentration in controls was 9 ng/mL (± 4 ng/mL SD; *n* = 19), mean total OCN in stage 3 CKD patients was 11 ng/mL (± 5 ng/mL SD; *n* = 20), and stage 4 CKD patients had a mean total OCN concentration of 22 ng/mL (± 7 ng/mL SD; *n* = 6). Total OCN was significantly increased in CKD stage 4 patients compared to controls (*p* < 0.001; Figure 1A), and in CKD patients as a whole (mean 14 ± 9 ng/mL) compared to controls (*p* < 0.05). Total OCN was significantly correlated to eGFR in the total population, in that there was a significant increase in total OCN concentrations when eGFR decreased (*n* = 43; *p* < 0.05; Figure 1B). Vascular calcification was detected in our sample population (mean age 76 years, Table 1) but calcium scores were not significantly different between CKD patients as a whole and controls, or CKD patients divided into stages compared to controls (Figure 1C). Total OCN was not correlated with calcium score, calcium density or pulse wave velocity within the CKD population or age-matched controls (Supplementary Figure 1).

### VSMC Morphology Is Lost in Cells Grown in Mineralisation Media

**Figure 2:**
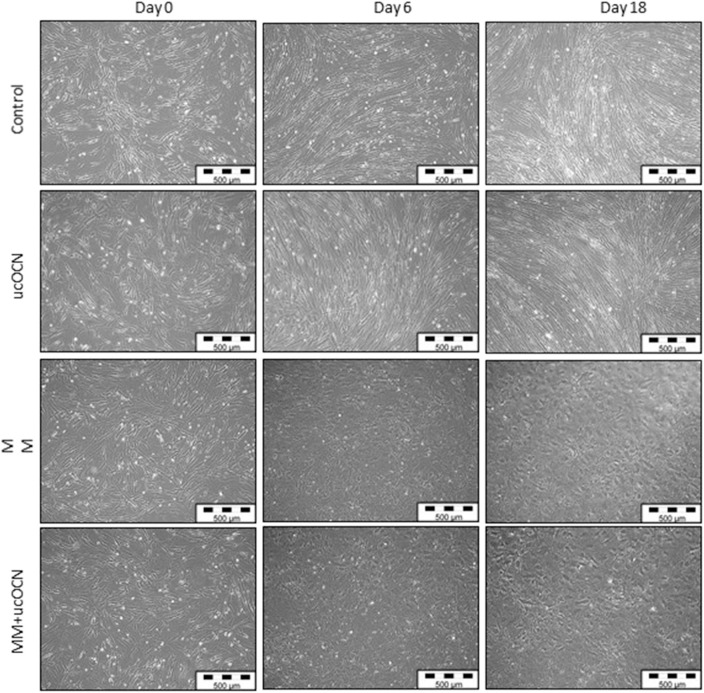
Human aortic smooth muscle cells (SMCs) were cultured in usual growth media (control) with or without ucOCN (10 ng/mL), or mineralisation inducing media (MM) with or without ucOCN (10 ng/mL). Photos (10X magnification) taken at days 0, 6, and 18 visualized by light microscopcopy. Control media treated cells maintained a classical SMC phenotype while MM treated cells displayed a differentiated synthetic phenotype distinctly different to control cells.

VSMCs maintained in usual smooth muscle cell growth media maintained classical spindle-shaped morphology of contractile smooth muscle cells throughout the experimental time points irrespective of treatment with or without ucOCN (Figure 2). In contrast, cells maintained in osteoblast MM acquired a more cobble-stone synthetic phenotype appearing by day 4 which progressed until the end of the experiment (Figure 2) (27). There were no visual differences between cells treated with MM with or without ucOCN.

**Figure 3:**
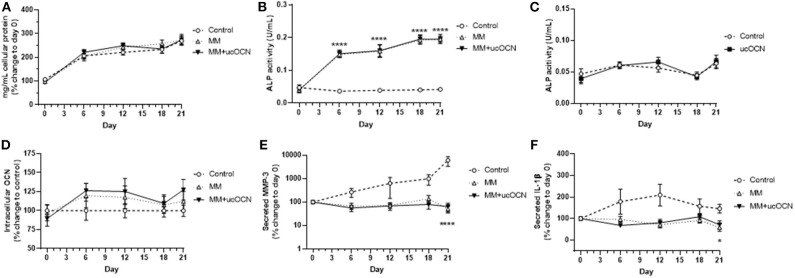
Human aortic smooth muscle cells were grown in usual growth media (control), or mineralisation inducing media (MM) with or without ucOCN (10 ng/mL). Total protein content **(A)**, ALP activity **(B)**, intracellular total osteocalcin **(D)**, secreted MMP-3 **(E)**, and secreted IL-1β **(F)** were measured at days 0, 6, 12, 18, and 21. ALP activity was also measured in control and control + ucOCN treated cells **(C)**. Data are represented by means with error bars representing SEM. Data were analyzed by two-way ANOVA using mixed effects analysis and Dunnett's test for multiple comparisons with *p < 0.05 and ****p < 0.0001 compared to control.

No significant differences were detected in protein content between any of the treatment groups and MM or ucOCN did not affect total protein content (Figure 3A).

### ALP Activity Is Increased in VSMCs Cultured in Mineralisation Media

ALP regulates pyrophosphate levels and promotes calcification by reducing pyrophosphate levels, as pyrophosphate is a potent inhibitor of calcification through inhibition of hydroxyapatite formation (28). Increased ALP activity is therefore used as a classical marker of transdifferentiated smooth muscle cells and of mineralisation and calcification. ALP activity was increased in MM treated cells with and without ucOCN, significantly apparent from day 6 with continued gradual increase until day 21 (Figure 3B; *p* < 0.001; days 6, 12, 18, and 21 compared to control). Cells treated with MM with and without ucOCN followed an identical trend, while cells maintained in normal smooth muscle cell media did not increase ALP activity throughout the experiment.

In a subset of experiments, cells were treated with normal smooth muscle cell media and ucOCN alone (10 ng/mL) to assess if ucOCN alone could stimulate calcification. There was no significant increase in ALP activity levels in cells treated with ucOCN which was undiscernible compared to control cells without ucOCN (Figure 3C) over 21 days.

### No Significant Differences Are Detected in Intracellular Osteocalcin Concentrations Between Treatments

Intracellular total osteocalcin appeared raised and fluctuated slightly over time in MM treated cells particularly at days 6 and 12 but this did not reach significance and there was no effect of ucOCN (Figure 3D). Extracellular secreted total osteocalcin, which is a marker of vascular smooth muscle cell osteoblastic differentiation, was not detected in any media samples after removing background levels already present in culture media over 21 days (data not shown).

### No Differences Are Detected in Secreted MMP-3 and IL-1β Concentration Between Cells Treated With or Without ucOCN

MMP-3 and IL-1β are associated with vascular calcification (29, 30). MMP-3 secretion increased with time in control media treated cells, and was increased compared to MM treated cells at day 21 (*p* < 0.0001; Figure 3E). MMP-3 secretion did not increase over time in MM treated cells, and no differences were detected between those treated with or without ucOCN (10 ng/mL). IL-1β secretion was also higher in control cells than those treated with MM (*p* < 0.05, day 21; Figure 3F). There were no differences between those treated with and without ucOCN (10 ng/ml).

### ucOCN Does Not Affect Alizarin Red Staining or Calcium Quantification

**Figure 4:**
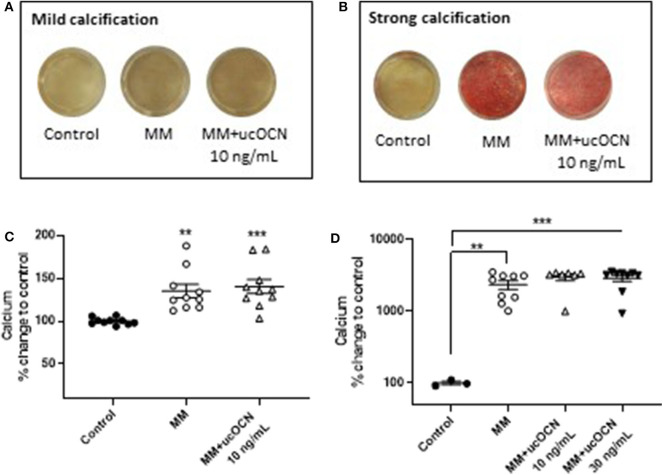
Human aortic smooth muscle cells (SMCs) were maintained in control media or mineralisation inducing media (MM). After 21 days, alizarin red staining was used to visualize calcification and calcium quantification was determined using a calcium assay. In some experiments SMCs mildly or moderately calcified **(A,C)** while in others they were strongly calcified **(B,D)**. Data are presented as means with error bars representing SEM. Data were analyzed by one-way ANOVA with **p < 0.01 and ***p < 0.001 compared to control.

Alizarin red staining was used to detect calcification. In half of the experiments performed, only mild calcification could be detected by alizarin red staining in MM treated cells after 21 days (Figure 4A), while in the other half of experiments strong calcification was detected (Figure 4B). A calcium quantification assay was performed which detected an increase in calcium in MM treated cells after 21 days, corresponding to the alizarin red staining (*p* < 0.01, Figure 4C). In the experiments which displayed strong calcification by alizarin red staining this was confirmed by large significant increases in calcium detected using the calcium assay (*p* < 0.01, Figure 4D). No calcium was detected in day 21 cells maintained in normal smooth muscle cell media. There were no differences between MM treated cells with or without ucOCN (10 or 30 ng/mL) in either the mild or strongly calcified cell experiments (Figure 4). In a subset of experiments, cells were maintained in normal smooth muscle cell media and treated with or without ucOCN alone (10 ng/mL). There was no detection of calcification visually or by quantification of alizarin red staining in these cells, and no differences were observed between those treated with or without ucOCN (data not shown). As a positive control, human osteoblasts (HOBs) were maintained in normal growth media or MM media. Both mild and moderate calcification was observed by visual alizarin red staining, quantification of alizarin red staining, and also by calcium quantification (Supplementary Figure 2).

### ucOCN Treatment Increases α-SMA Expression While Runx2 Is Not Affected

**Figure 5:**
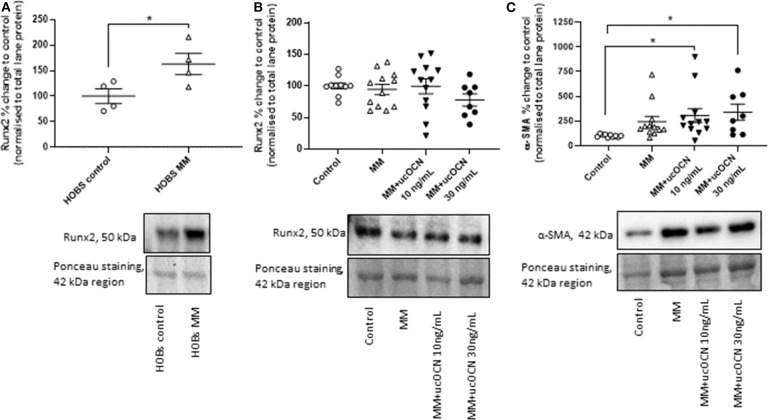
Human aortic smooth muscle cells (SMCs) were cultured in usual growth media (control), or mineralisation media (MM) with or without ucOCN (10 or 30 ng/mL). **(A)** Human osteoblasts (HOBs) were also cultured in control or MM and Runx2 expression was measured by western blotting. Expression of Runx2 **(B)** and α-SMA **(C)** in SMCs were measured by western blotting on day 21. Data are presented as means with error bars representing SEM. Data were analyzed by one-way ANOVAs or t-tests with *p < 0.05.

Runx2/Cbfa1 is a master transcriptional regulator essential for ossification and is classically used as an osteogenic marker. α-SMA is a classical smooth muscle cell marker and is usually decreased in osteogenic differentiated VSMCs. Runx2 expression was not increased in our MM treated cells and there were no significant differences between groups (Figure 5B). However, in our positive control experiment in HOBs, runx2 was significantly increased in MM treated cells as expected (Figure 5A, *p* < 0.05 compared to control). Additionally, smooth muscle cells treated with MM and ucOCN (10 and 30 ng/mL) displayed an unexpected significantly increased expression of α-SMA compared to control (Figure 5C; *p* < 0.05). Sox9 expression was not detectable in our samples (data not shown).

## Discussion

Due to conflicting and limited epidemiological and *in vitro* data on the relationship between OCN and vascular calcification (15, 16), we aimed to investigate for the first time in human aortic smooth muscle cells whether ucOCN affects the speed or extent of vascular calcification, and to further assess any relationship between plasma total OCN and CKD patients. This study found a significant relationship between circulating total OCN concentrations and renal function (eGFR) of CKD patients, but found no *in vitro* evidence of an active role of ucOCN at physiological and pathological concentrations during vascular calcification as assessed over 21 days.

In our data, circulating total OCN concentrations were significantly higher in CKD patients compared to age-matched control patients, and were inversely correlated with eGFR. This is consistent with previous studies in pre-dialysis CKD patients which also found a negative relationship between OCN and GFR (31, 32). Our circulating concentrations were similar to those reported elsewhere for CKD haemodialysis patients (33). Levels of OCN-positive circulating endothelial progenitor cells have been found to be increased in haemodialysis patients compared to controls (34). Total OCN was not however correlated with calcification scoring or carotid-femoral pulse wave velocity, a measure of arterial stiffness, in our group as a whole. There were no significant differences between our control group and CKD group in cardiovascular measurements but perhaps if more prominent calcification was present in our sample a correlation may have been identified. There also may be a distinct difference in the role of ucOCN vs. carboxylated OCN (cOCN), however these different forms are not routinely measured and could not be investigated (35). The patients assessed were stage 3 and stage 4 CKD patients, and it is possible that stage 5 CKD patients would have higher concentrations of circulating OCN, and thus a relationship with calcification may become apparent. In our previous meta-analysis of the relationship between OCN and vascular calcification, no conclusion could be drawn on the relationship due to heterogeneous data and conflicting results (15). In the present study, the results do not promote the viewpoint of OCN having a causal, active effect on vascular calcification.

Our *in vitro* findings reject a hypothesis for a direct involvement of ucOCN in vascular calcification. After 21 days, cells cultured in mineralisation inducing media had calcium depositions and increased ALP activity, alongside distinctly altered morphology. Control cells did not have any calcium detected, nor increases in ALP, and retained classical vascular smooth muscle cell morphology. The addition of ucOCN at two concentrations (based on levels measured in our CKD patients) did not affect the endpoints examined, suggesting that ucOCN does not have a direct role in vascular calcification but is rather a by-product of osteogenic transdifferentiation. Furthermore, addition of ucOCN alone to control cells did not increase ALP activity nor induce calcification. It is important to note that within our experiments, assessing endpoints individually between those that displayed strong calcification, and those that showed mild calcification, also did not reveal an effect of ucOCN. Despite components of the osteogenic fingerprint of transdifferentiated vascular smooth muscle cells being observed (such as increased ALP and calcium), a couple of unexpected results were also obtained. Differentiated VSMCs displayed an increased expression of α-SMA when treated with ucOCN, which is usually decreased in osteogenic differentiated cells, and no changes in Runx2 expression were observed, which is usually increased (6). It is noteworthy that cells treated with mineralisation media alone did not significantly increase α-SMA expression, only with the addition of ucOCN did the increase in expression become significant. Combined with the lack of change in Runx2 expression, it could be speculated that ucOCN is possibly inhibiting differentiation (as α-SMA is a classical VSMC marker). Although these findings were unexpected it is widely appreciated that VSMCs possess remarkable phenotypic flexibility and it may simply transpire that the cells in these experiments represent an earlier phenotype before complete differentiation and widespread calcification. The mechanisms of vascular calcification and endochondral transitioning is a complex research area that is not fully understood. The increase in α-SMA however is interesting and it has been reported elsewhere that a higher expression has been observed in mineralised nodules in aortic VSMCs (36). Of note, in calcific aortic stenosis, the smooth muscle cell phenotype remains, and in myofibroblast differentiation and calcification, Runx2 and α-SMA are dually increased in the osteogenic/osteoblastic phenotype (37, 38). As multiple sub-populations of VSMCs exist, including synthetic, contractile, and particularly calcifying prone cell phenotypes, we recommend other sources of VSMCs are investigated with ucOCN to confirm our findings (39, 40). Lastly, it may transpire that cOCN, which has a higher affinity for calcium ions, may be more relevant in vascular calcification and further studies should also address this specifically.

In mildly-calcified cells, ucOCN did not increase calcification, and in strongly-calcified cells ucOCN did not decrease calcification. Previously, OCN has been proposed to be involved in the regulation of arterial calcification as it is present in calcified regions in humans (15). In a rabbit *in vivo* model, OCN was detected in 8- and 14-day calcified structures but not earlier, suggesting OCN may not be involved in the initiation of calcification but rather later regulation (41). Elsewhere, OCN levels increased with osteogenic differentiation in two different mice cell lines, chondrocytes and VSMCs, and when overexpressed, OCN functioned as a stimulator of differentiation and mineralization, upregulating Sox9, Runx2, collagen type X, ALP, proteoglycans, and mineral content in both of these cell types (16). In matrix GLA protein null mice (MGP^−/−^) OCN did not display any anti-mineralisation function in arteries nor did over-expression of OCN in osteoblasts inhibit normal mineralisation in bone (13). A correlation between aortal calcification and elevation of OCN in 1,25(OH)2D3-treated rats, which was hampered by OCN siRNA silencing, has also been shown (16). Importantly however, these studies have not been performed in humans or human cells, thus this study is the first to examine the effects of ucOCN in human VSMCs.

Our examination of IL-1β and MMP-3 secretion revealed some interesting insights. IL-1β has emerged in recent years as a potential stimulator of vascular calcification for example by increasing ALP activity, and has been proposed as a marker of inflammatory calcification (29, 42). In contrast, we found significantly increased secretion of IL-1β over time in control cells only, compared to mineralisation media treated cells. It may transpire that immune cell secreted IL-1β contributes to calcification of VSMCs, but IL-1β secreted from VSMCs themselves does not induce calcification. At least in our experiments, VSMC secreted IL-1β may even be protective, as calcified cells did not have increased levels. Differential cell specific actions of IL-1β have previously been demonstrated, for example addition of IL-1β to chondrocytes *in vitro* was shown to inhibit ALP activity (42). Similarly, MMP-3 secretion was increased over time in control cells only, particularly apparent at day 21. MMP-3 is required for the degradation of the extracellular matrix and has been associated with vascular calcification, particularly within atherosclerotic plaques (30). However, our results would suggest that MMP-3 secretion from VSMCs may be protective against calcification as control cells did not calcify. This may be due to differences in models of calcification used, and more pro-inflammatory and atherosclerotic models may show different effects of IL-1β and MMP-3. Importantly however, there was no inhibitory or stimulatory effect of ucOCN on either IL-1β or MMP-3 secretion.

## Conclusions

OCN has been consistently detected in vascular calcification plaques. However, circulating total OCN levels were not correlated with calcification or pulse wave velocity in our study population. ucOCN over 21 days did not, in either mild or strong calcification instances, increase the speed or extent of osteogenic calcification of human VSMCs, nor showed any inhibitory effects. The major limitations of our study include measurement of total OCN only in our human population, and studying a population which had a low level of calcification. Additionally, we only assessed *in vitro* the affects of one circulating form of osteocalcin; ucOCN. The results presented in this study suggest that ucOCN is likely not an active contributor to calcification, but its consistent presence detected in vascular calcification reported in relevant literature may indicate that it is simply a resulting product of the process of calcification and trans-differentiation of osteogenic vascular cells. Further investigations of other circulating forms of OCN and in other vascular cell types and conditions are recommended to confirm these findings.

## Data Availability Statement

The datasets generated for this study are available on request to the corresponding author.

## Ethics Statement

The study was originally approved by the Local Regional Ethics Committee and all patients gave written informed consent.

## Author Contributions

SM and SO'S: study design. SM, SO'S, CM, and SJ: study conduct. SM: data analysis and drafting manuscript. All authors: data interpretation, revising manuscript content, and approving final version of manuscript.

## Conflict of Interest

The authors declare that the research was conducted in the absence of any commercial or financial relationships that could be construed as a potential conflict of interest.

## References

[1] Wayhs R Zelinger A Raggi P. High coronary artery calcium scores pose an extremely elevated risk for hard events. J Am Coll Cardiol. (2002) 39:225–30. 10.1016/S0735-1097(01)01737-5 11788211

[2] Arad Y Spadaro LA Goodman K Newstein D Guerci AD. Prediction of coronary events with electron beam computed tomography. J Am Coll Cardiol. (2000) 36:1253–60. 10.1016/S0735-1097(00)00872-X 11028480

[3] Bowman MAH McNally EM. Genetic pathways of vascular calcification. Trends Cardiovasc Med. (2012) 22:93–8. 10.1016/j.tcm.2012.07.002 23040839 PMC3466440

[4] Nakamura S Ishibashi-Ueda H Niizuma S Yoshihara F Horio T Kawano Y. Coronary calcification in patients with chronic kidney disease and coronary artery disease. Clin J Am Soc Nephrol. (2009) 4:1892–900. 10.2215/CJN.04320709 19833908 PMC2798876

[5] Demer LL Tintut Y. Vascular calcification: pathobiology of a multifaceted disease. Circulation. (2008) 117:2938–48. 10.1161/CIRCULATIONAHA.107.743161 18519861 PMC4431628

[6] Evrard S Delanaye P Kamel S Cristol JP Cavalier E. Vascular calcification: from pathophysiology to biomarkers. Clin Chim Acta. (2015) 438:401–14. 10.1016/j.cca.2014.08.034 25236333

[7] Steitz SA Speer MY Curinga G Yang HY Haynes P Aebersold R. Smooth muscle cell phenotypic transition associated with calcification: upregulation of Cbfa1 and downregulation of smooth muscle lineage markers. Circ Res. (2001) 89:1147–54. 10.1161/hh2401.101070 11739279

[8] Leopold JA. Vascular calcification: mechanisms of vascular smooth muscle cell calcification. Trends Cardiovasc Med. (2015) 25:267–74. 10.1016/j.tcm.2014.10.021 25435520 PMC4414672

[9] Hauschka PV Lian JB Gallop PM. Direct identification of the calcium-binding amino acid, gamma-carboxyglutamate, in mineralized tissue. Proc Natl Acad Sci USA. (1975) 72:3925–9. 10.1073/pnas.72.10.3925 1060074 PMC433109

[10] Hauschka PV Lian JB Cole DE Gundberg CM. Osteocalcin and matrix Gla protein: vitamin K-dependent proteins in bone. Physiol Rev. (1989) 69:990–1047. 10.1152/physrev.1989.69.3.990 2664828

[11] Tyson KL Reynolds JL McNair R Zhang Q Weissberg PL Shanahan CM. Osteo/chondrocytic transcription factors and their target genes exhibit distinct patterns of expression in human arterial calcification. Arterioscler Thromb Vasc Biol. (2003) 23:489–94. 10.1161/01.ATV.0000059406.92165.31 12615658

[12] Ducy P Desbois C Boyce B Pinero G Story B Dunstan C. Increased bone formation in osteocalcin-deficient mice. Nature. (1996) 382:448–52. 10.1038/382448a0 8684484

[13] Murshed M Schinke T McKee MD Karsenty G. Extracellular matrix mineralization is regulated locally; different roles of two gla-containing proteins. J Cell Biol. (2004) 165:625–30. 10.1083/jcb.200402046 15184399 PMC2172384

[14] Hunter GK Hauschka PV Poole AR Rosenberg LC Goldberg HA. Nucleation and inhibition of hydroxyapatite formation by mineralized tissue proteins. Biochem J. (1996) 317:59–64. 10.1042/bj3170059 8694787 PMC1217486

[15] Millar SA Patel H Anderson SI England TJ O'Sullivan SE. Osteocalcin, vascular calcification, and atherosclerosis: a systematic review and meta-analysis. Front Endocrinol. (2017) 8:183. 10.3389/fendo.2017.00183 28824544 PMC5534451

[16] Idelevich A Rais Y Monsonego-Ornan E. Bone Gla protein increases HIF-1alpha-dependent glucose metabolism and induces cartilage and vascular calcification. Arterioscler Thromb Vasc Biol. (2011) 31:e55–71. 10.1161/ATVBAHA.111.230904 21757657

[17] John SG Owen PJ Harrison LEA Szeto CC Lai KB Li PKT. The impact of antihypertensive drug therapy on endotoxemia in elderly patients with chronic kidney disease. Clin J Am Soc Nephrol. (2011) 6:2389–94. 10.2215/CJN.11211210 21852662 PMC3359560

[18] McIntyre CW Harrison LE Eldehni MT Jefferies HJ Szeto CC John SG. Circulating endotoxemia: a novel factor in systemic inflammation and cardiovascular disease in chronic kidney disease. Clin J Am Soc Nephrol. (2011) 6:133–41. 10.2215/CJN.04610510 20876680 PMC3022234

[19] Sigrist M Bungay P Taal MW McIntyre CW. Vascular calcification and cardiovascular function in chronic kidney disease. Nephrol Dial Transplant. (2006) 21:707–14. 10.1093/ndt/gfi236 16263735

[20] Henstock JR Ruktanonchai UR Canham LT Anderson SI. Porous silicon confers bioactivity to polycaprolactone composites *in vitro*. J Mater Sci Mater Med. (2014) 25:1087–97. 10.1007/s10856-014-5140-5 24398914

[21] Anderson SI Downes S Perry CC Caballero AM. Evaluation of the osteoblast response to a silica gel **in vitro. J Mater Sci Mater Med. (1998) 9:731–5. 10.1023/A:1008955002955 15348931

[22] Huang J Di Silvio L Wang M Tanner KE Bonfield W. *In vitro* mechanical and biological assessment of hydroxyapatite-reinforced polyethylene composite. J Mater Sci Mater Med. (1997) 8:775–9. 15348789 10.1023/a:1018516813604

[23] Millar SA Anderson SI O'Sullivan ES. Human vascular cell responses to the circulating bone hormone osteocalcin. J Cell Physiol. (2019) 234:21039–48. 10.1002/jcp.28707 31026070 PMC6767466

[24] Smith PK Krohn RI Hermanson GT Mallia AK Gartner FH Provenzano MD. Measurement of protein using bicinchoninic acid. Anal Biochem. (1985) 150:76–85. 10.1016/0003-2697(85)90442-7 3843705

[25] Gitelman H An improved automated procedure for the determination of calcium in biological specimen. Anal Biochem. (1967) 18:521–31. 10.1016/0003-2697(67)90110-8

[26] Moritz CP Tubulin or not tubulin: heading toward total protein staining as loading control in western blots. Proteomics. (2017) 17. 10.1002/pmic.201600189 28941183

[27] Durham AL Speer MY Scatena M Giachelli CM Shanahan CM Role of smooth muscle cells in vascular calcification: implications in atherosclerosis and arterial stiffness. Cardiovasc Res. (2018) 114:590–600. 10.1093/cvr/cvy010 29514202 PMC5852633

[28] O'Neill WC. Pyrophosphate, alkaline phosphatase, and vascular calcification. Circ Res. (2006) 99:e2. 10.1161/01.RES.0000234909.24367.a9 16857967

[29] Shobeiri N Bendeck MP. Interleukin-1beta is a key biomarker and mediator of inflammatory vascular calcification. Arterioscler Thromb Vasc Biol. (2017) 37:179–80. 10.1161/ATVBAHA.116.308724 28122774

[30] Galis ZS Sukhova GK Lark MW Libby P. Increased expression of matrix metalloproteinases and matrix degrading activity in vulnerable regions of human atherosclerotic plaques. J Clin Invest. (1994) 94:2493–503. 10.1172/JCI117619 7989608 PMC330083

[31] Yamada S Inaba M Kurajoh M Shidara K Imanishi Y Ishimura E. Utility of serum tartrate-resistant acid phosphatase (TRACP5b) as a bone resorption marker in patients with chronic kidney disease: independence from renal dysfunction. Clin Endocrinol. (2008) 69:189–96. 10.1111/j.1365-2265.2008.03187.x 18221403

[32] Jiang JQ Lin S Xu PC Zheng ZF Jia JY. Serum osteoprotegerin measurement for early diagnosis of chronic kidney disease-mineral and bone disorder. Nephrology. (2011) 16:588–94. 10.1111/j.1440-1797.2011.01481.x 21649792

[33] Kuzniewski M Fedak D Dumnicka P Kapusta M Stepien E Chowaniec E. Carboxylated and intact osteocalcin predict adiponectin concentration in hemodialyzed patients. Ren Fail. (2016) 38:451–7. 10.3109/0886022X.2016.1138830 26822199

[34] Cianciolo G Capelli I Cappuccilli M Scrivo A Donadei C Marchetti A. Is chronic kidney disease-mineral and bone disorder associated with the presence of endothelial progenitor cells with a calcifying phenotype? In Clin Kidney J. (2017) 10:389–96. 10.1093/ckj/sfw145 28616217 PMC5466108

[35] Lee AJ Hodges S Eastell R Measurement of osteocalcin. Ann Clin Biochem. (2000) 37:432–46. 10.1177/000456320003700402 10902858

[36] Proudfoot D Skepper JN Shanahan CM Weissberg PL. Calcification of human vascular cells *in vitro* is correlated with high levels of matrix Gla protein and low levels of osteopontin expression. Arterioscler Thromb Vasc Biol. (1998) 18:379–88. 10.1161/01.ATV.18.3.379 9514406

[37] Latif N Sarathchandra P Chester AH Yacoub MH. Expression of smooth muscle cell markers and co-activators in calcified aortic valves. Eur Heart J. (2015) 36:1335–45. 10.1093/eurheartj/eht547 24419809

[38] Hjortnaes J Goettsch C Hutcheson JD Camci-Unal G Lax L Scherer K. Simulation of early calcific aortic valve disease in a 3D platform: a role for myofibroblast differentiation. J Mol Cell Cardiol. (2016) 94:13–20. 10.1016/j.yjmcc.2016.03.004 26996755 PMC4906202

[39] Watson KE Boström K Ravindranath R Lam T Norton B Demer LL. TGF-beta 1 and 25-hydroxycholesterol stimulate osteoblast-like vascular cells to calcify. J Clin Invest. (1994) 93:2106–13. 10.1172/JCI117205 8182141 PMC294336

[40] Trion A van der Laarse A. Vascular smooth muscle cells and calcification in atherosclerosis. Am Heart J. (2004) 147:808–14. 10.1016/j.ahj.2003.10.047 15131535

[41] Gadeau AP Chaulet H Daret D Kockx M Daniel-Lamaziere JM Desgranges C. Time course of osteopontin, osteocalcin, and osteonectin accumulation and calcification after acute vessel wall injury. J Histochem Cytochem. (2001) 49:79–86. 10.1177/002215540104900108 11118480

[42] Lencel P Delplace S Pilet P Leterme D Miellot F Sourice S. Cell-specific effects of TNF-alpha and IL-1beta on alkaline phosphatase: implication for syndesmophyte formation and vascular calcification. Lab Invest. (2011) 91:1434–42. 10.1038/labinvest.2011.83 21555997

