# Supplementary figures and images for: An Investigation Into the Role of Osteocalcin in Human Arterial Smooth Muscle Cell Calcification
Source: Front Endocrinol (Lausanne). 2020 Jun 10;11:369. doi: 10.3389/fendo.2020.00369 (PMC7298126; doi:10.3389/fendo.2020.00369)

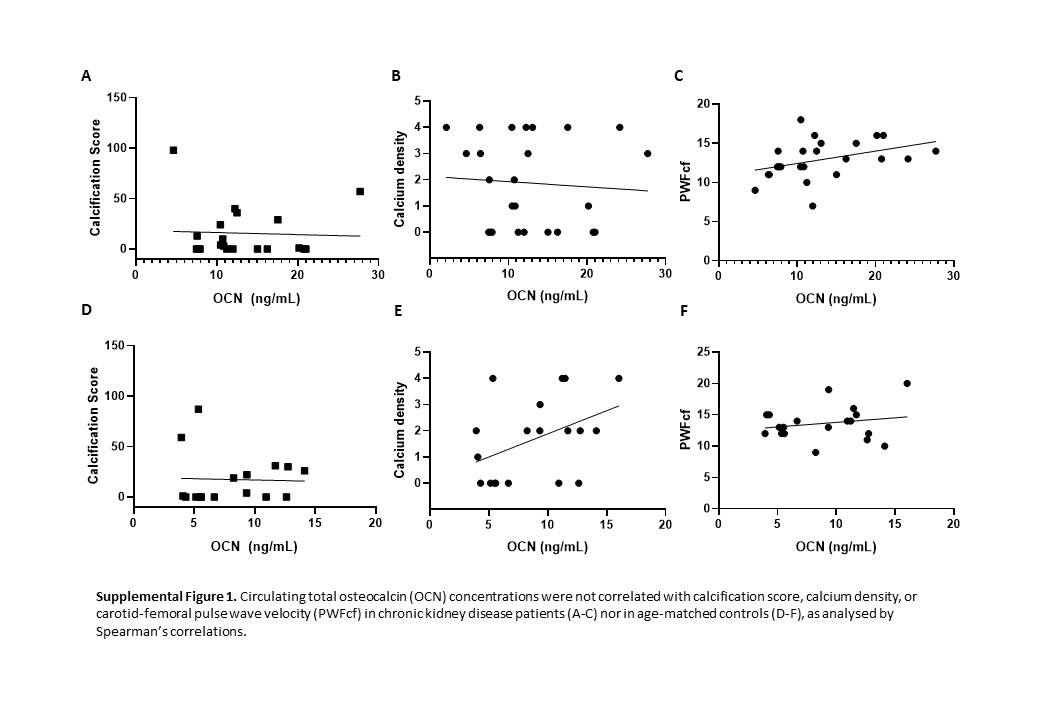

Supplement: Supplementary file 1 [file Image_1.jpeg]

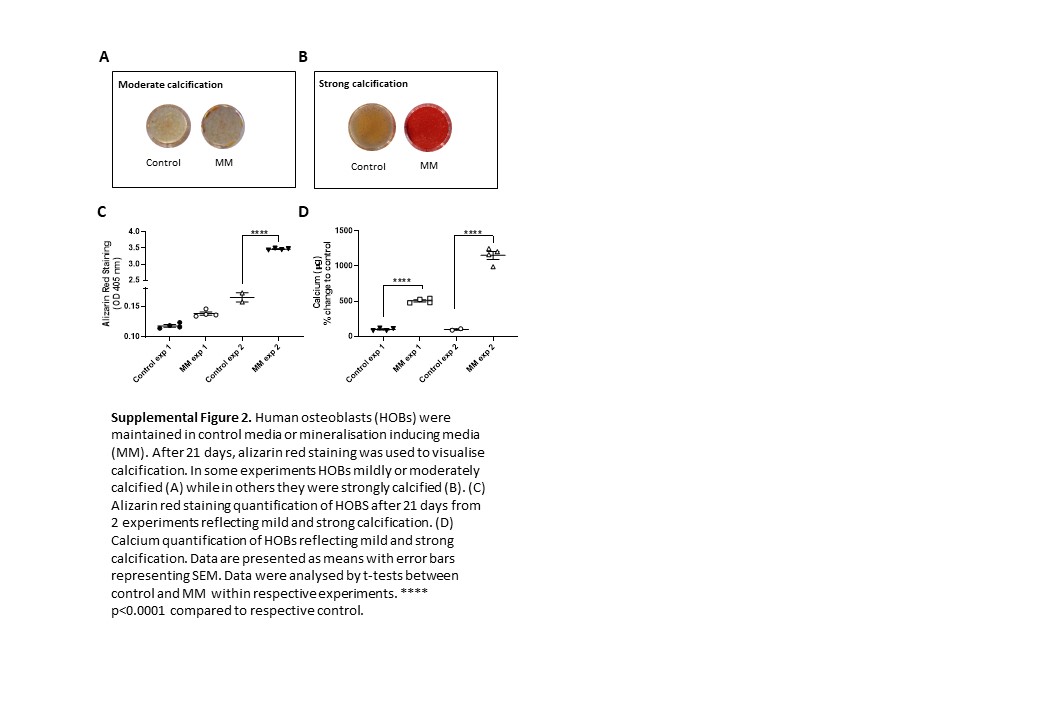

Supplement: Supplementary file 2 [file Image_2.jpeg]
